# High Genetic Diversity and Distinctiveness of Rear-Edge Climate Relicts Maintained by Ancient Tetraploidisation for *Alnus glutinosa*

**DOI:** 10.1371/journal.pone.0075029

**Published:** 2013-09-30

**Authors:** Olivier Lepais, Serge D. Muller, Samia Ben Saad-Limam, Mohamed Benslama, Laila Rhazi, Djamila Belouahem-Abed, Amina Daoud-Bouattour, Amor Mokhtar Gammar, Zeineb Ghrabi-Gammar, Cécile Fanny Emilie Bacles

**Affiliations:** 1 Biological and Environmental Sciences, School of Natural Sciences, University of Stirling, Stirling, United Kingdom; 2 INRA, UMR 1224, Ecologie Comportementale et Biologie des Populations de Poissons, Saint Pée sur Nivelle, France; 3 Univ Pau & Pays Adour, UMR 1224, Ecologie Comportementale et Biologie des Populations de Poissons, Anglet, France; 4 Université Montpellier 2, CNRS, UMR 5554, Institut des Sciences de l’Evolution, Montpellier, France; 5 Université de Tunis El Manar, Faculté des Sciences de Tunis, Département de Biologie, Tunis, Tunisie; 6 Université de Manouba, UR Biogéographie, Climatologie Appliquée et Dynamique Erosive, Faculté des Lettres des Arts et des Humanités de Manouba, Manouba, Tunisie; 7 Laboratoire de Biologie Végétale et Environnement, Université Badji Mokhtar, Annaba, Algérie; 8 Université Hassan II Casablanca, Faculté des Sciences Aïn Chock, Laboratoire d’Ecologie Aquatique et Environnement, Casablanca, Maroc; 9 Institut National de Recherche forestière, Station de Recherche, El Kala, Algérie; 10 Université de Carthage, Institut National Agronomique de Tunisie, Tunis, Tunisie; CNRS/Université Joseph-Fourier, France

## Abstract

Populations located at the rear-edge of a species’ distribution may have disproportionate ecological and evolutionary importance for biodiversity conservation in a changing global environment. Yet genetic studies of such populations remain rare. This study investigates the evolutionary history of North-African low latitude marginal populations of *Alnus glutinosa* Gaertn., a European tree species that plays a significant ecological role as a keystone of riparian ecosystems. We genotyped 551 adults from 19 populations located across North Africa at 12 microsatellite loci and applied a coalescent-based simulation approach to reconstruct the demographic and evolutionary history of these populations. Surprisingly, Moroccan trees were tetraploids demonstrating a strong distinctiveness of these populations within a species otherwise known as diploid. Best-fitting models of demographic reconstruction revealed the relict nature of Moroccan populations that were found to have withstood past climate change events and to be much older than Algerian and Tunisian populations. This study highlights the complex demographic history that can be encountered in rear-edge distribution margins that here consist of both old stable climate relict and more recent populations, distinctively diverse genetically both quantitatively and qualitatively. We emphasize the high evolutionary and conservation value of marginal rear-edge populations of a keystone riparian species in the context of on-going climate change in the Mediterranean region.

## Introduction

A poleward shift in the geographical distribution of many species is expected as one of the major consequences of on-going global climate change [1]. The intensity of this distributional shift will depend largely on the response of populations situated at the margin of a species’ distribution which has prompted theoretical and empirical research efforts of the past decade to focus on dynamics of leading-edge populations, located at high latitude or high altitude, at the forefront of colonisation [2], [3], [4]. However, it has recently been argued that low altitude low latitude marginal populations located at the current rear-edge of a species’ distribution have not attracted enough attention given their disproportionate ecological and evolutionary importance for biodiversity conservation in a changing global environment [5], [6]. In this context, the aim of this empirical study is to describe the evolutionary history of contemporary North-African rear-edge populations of black alder, *Alnus glutinosa* Gaertn., a monoecious self-incompatible wind-dispersed European tree species with fruits dispersed by wind at small distance and by water at longer distance and which acts as a keystone of riparian ecosystems [7]. With such insight of the resilience of current rear-edge populations to previous range contractions and expansions, we aim to clarify their conservation priority status and predictive importance in the context of on-going climate change [6].

Although different responses to past climatic events have been reported for species with contrasted ecological characteristics [8], [9], empirical investigations of rear-edge populations of species acting as keystone of riparian ecosystems remain scarce [10] despite their recognised ecological importance. A large body of evidence shows that many European temperate tree species survived the Last Glacial Maximum (LGM) of the Quaternary era in refugia. Those genetic diversity “hot spots” have acted as source for subsequent species expansion and have shaped their current range-wide genetic structure [11], [12]. The spread of *A. glutinosa* over most of Europe was achieved in the early Holocene, as shown by pollen and macrofossil records which indicate its presence in Southern Switzerland by 14,000 cal. BP [13], in Northern French Alps by 12,300 cal. BP [14], in most of the British Isles by 10,000 cal. BP [15], in Northern Germany by 9000 cal. BP [16] and in Fennoscandia by 8800-8000 cal. BP [17] approximately. Pollen records have allowed the identification of LGM refugia for *A. glutinosa* in central and eastern Europe [18] and around the Mediterranean basin: in Corsica [19], in the Iberian [20], [21], Italian [22], [23], and Greek [24] peninsulas, and in Turkey [25] and south, in Tunisia [26], [27] and Algeria [28]. In an extensive phylogenetic analysis of chloroplast DNA variation of European populations of *A. glutinosa*, [29] confirmed the location of LGM refugia in southern Europe and revealed that most of Northern and Central Europe has been recolonised from refugia located in the Carpathian Mountains with other refugia for Northern Europe likely to have been located east of Alps. However, King & Ferris [29] pointed out that their sampling may have left out some Southern populations, such as North African rear-edge populations, of potential significance to the postglacial expansion of the species.

Stable rear-edge populations [5] are defined as climate relicts that have persisted across not just one but several cold and warm stages of the Quaternary, and may have remained isolated from more centrally located LGM refugia. Thanks to their long-term persistence and geographic isolation, climate relicts are predicted to be characterized by disproportionately high levels of regional genetic diversity and genetic distinctiveness and as such should be given highest priority for conservation [5]. The importance of rear-edge climate relicts is all the more significant for global change research that these populations have withstood past climate changes in pockets of locally favourable habitat in otherwise regionally unsuitable environmental conditions. They constitute a remarkable laboratory in which population history reconstruction can reveal past species response to climate change thus helping to forecast contemporary species response to present and future climate changes [6].

North-African rear-edge populations of *A. glutinosa* are good candidate climate relicts. Firstly, pollen records indicate a long-term presence of *A. glutinosa* in North Africa [26], [27], [28], [30]. Secondly, as pointed out by Hampe & Petit [5] stable rear-edge climate relicts are most likely to be found in heterogeneous landscapes in mountainous or riparian ecosystems that may buffer global environmental impacts locally by presenting opportunity for micro-local refugia (e.g. slop orientation) and because the physical environment present some inertia to change in atmospheric temperature (e.g. water availability). The current distribution of this riparian species in North Africa is extremely scattered, confined to a handful of small populations in difficult to access mountainous and coastal streams and peatlands that match the expected ecological features of climate relicts. Nonetheless, such riparian habit can also be expected to bring instability in species’ distribution as species occurring in riparian habitats are, by nature, fragmented in small populations across the landscape and conversely can be expected to respond strongly to climate change in dry periods which may result in local extirpation and range shift [31].

Contemporary North-African populations clearly lay at the rear-edge of the current distribution of *A. glutinosa*, however their evolutionary history remains unclear. In this paper, we consider three alternative hypothetical scenarios. The first one hypothesises that North-African populations constituted LGM refugia that contributed to postglacial recolonisation of Europe. A second scenario considers that North-African populations are pre-LGM stable rear-edge climate relicts that did not contribute to postglacial recolonisation. In these two scenarios, populations would be expected to display a relatively low genetic diversity within populations but high regional genetic diversity and genetic differentiation. However expected differential characteristics of climate relicts are high genetic distinctiveness resulting from a longer term isolation [5]. A third possible scenario would be due to the rarity and patchiness of the riparian habitats in North Africa, and *A. glutinosa* population dynamics would follow a metapopulation process where local populations are transient. This third scenario would consider the current North-African distribution to represent a subset of the genetic diversity of the core post-LGM distribution.

In order to discriminate between those alternative hypotheses and clarify the conservation status of *A. glutinosa* in North Africa, we use recently developed species-specific nuclear microsatellite markers [32] and coalescence-based demographic history reconstruction methods [33] to (1) estimate and compare levels of population genetic diversity in populations sampled in Morocco, Algeria, Tunisia; (2) describe the level of genetic differentiation among these populations, and (3) fit several demographic models of population history to the sampled populations and provide statistical support to interpretations of evolutionary history of *A. glutinosa* in the region from best-fitted models. Our results demonstrate the complex demographic history that can be encountered in rear-edge populations and contribute to emphasize the high evolutionary and conservation value of rear-edge populations in the context of future climate change.

## Materials and Methods

### Ethics Statement

No permits were required for the field study as only a few leaves of tree were collected with no effect significant to individual and ecosystem health. The sampled species is not protected nor is the accessed land.

### Study Populations and Plant Material Collection

**Figure 1:**
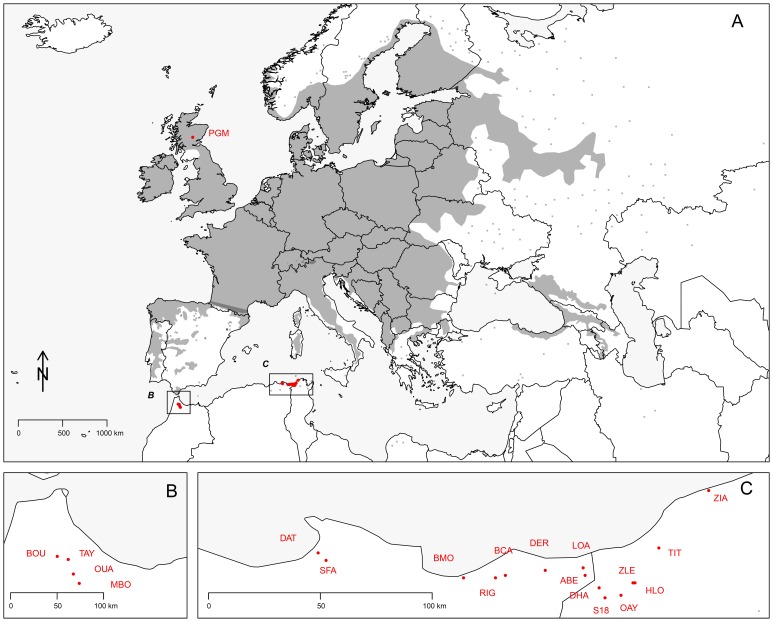
Distribution area (A) and sampling locations (B and C) of *A. glutinosa*. Small grey squares represent fragmented distribution at the distribution area margin while grey area represent more continuous distribution according to data compiled and released by the EUFORGEN Network. Population codes refer to Table 1.

In April-May 2010, leaf material was collected from a total of 551 adult trees with an average of 30 individuals sampled from 19 *Alnus glutinosa* populations throughout the current known species distribution in North African and persisting in locally wet habitats in pockets of riparian forests, small floodplains and alder carrs, located from sea level upwards to altitudes nearing 1000 m (Table 1). 15 of these populations were located along a 218 km longitudinal stretch in northwestern Tunisia and northeastern Algeria within a range of 6 to 76 km of each others (Fig. 1). Material was also collected from four riparian populations located within 12 to 16 km of each others in the mountains of the western Rif in Northern Morocco, isolated from the nearest Algerian population by a distance of 1400 km (Fig. 1). Collected leaf material was dried and kept in silica gel beads at room temperature for long-term storage.

**Table 1:** Geographic characteristics and nuclear genetic diversity parameters estimated for 20 *A. glutinosa* populations.

Sampling site	Code	Location	Country	Alt (m)	N	Nal1a	Nal2a	Nal3a	Nal4a	Ploidy#	Hs	rHs
Oued Ziatine	ZIA	Cap Serrat	Tunisia	10	30	0	30	0	0	2	0.54	0.52
Oued Titria	TIT	Ouchtata	Tunisia	82	30	0	30	0	0	2	0.57	0.57
Oued Zlezel	ZLE	Ain Draham	Tunisia	355	30	0	30	0	0	2	0.59	0.54
Oued Hfor Loussif	HLO	Ain Draham	Tunisia	355	30	0	30	0	0	2	0.57	0.61
Oued Ouled Ayed	OAY	Ain Draham	Tunisia	484	30	0	30	0	0	2	0.58	0.56
Sources du 18e	S18	Ain Draham	Tunisia	750	30	0	30	0	0	2	0.57	0.54
Oued Dhalma	DHA	Ain Draham	Tunisia	395	31	0	31	0	0	2	0.52	0.53
Ain Bergougaia	ABE	Oum Tebboul	Algeria	306	12	0	12	0	0	2	0.48	0.48
Laouledj	LAO	El Kala	Algeria	29	30	0	30	0	0	2	0.51	0.47
Demnat Errihane	DER	El Kala	Algeria	30	30	0	30	0	0	2	0.51	0.54
Righia	RIG	Berrihane	Algeria	30	30	0	30	0	0	2	0.54	0.49
Berrihane Café	BCA	Berrihane	Algeria	12	30	0	30	0	0	2	0.50	0.49
Bou Mohacene	BMO	Ben Mehidi	Algeria	6	30	0	30	0	0	2	0.49	0.44
Sidi Freitis	SFA	Guerbes	Algeria	15	30	0	30	0	0	2	0.54	0.53
Demnat Ataoua	DAT	Guerbes	Algeria	16	30	0	30	0	0	2	0.54	0.54
Bouztate	BOU	Bab Taza	Morocco	1013	30	0	0	3	27	4	0.78	0.76
Oued Ouara	OUA	Chefchaouen	Morocco	272	30	0	1	3	26	4	0.77	0.70
Oued Moulay Boucheta	MBO	Chefchaouen	Morocco	403	30	0	0	1	29	4	0.77	0.70
Tayenza	TAY	Tayenza	Morocco	974	28	0	0	1	27	4	0.75	0.73
Logierait	PGM	Perthshire	Scotland	70	36	0	36	0	0	2	0.67	0.63

Population codes refer to the ones used in Fig. 1. For comparison, characteristics are also given for one previously studied postglacial European population (PGM) [32]. Alt: Altitude, N: number of individuals sampled for molecular analyses. Genetic diversity parameters are based on genotyping of 11 microsatellite markers described in [32]. N_al1a_: number of individuals with at most one allele at one or more loci, N_al2a_: number of individuals with at most two alleles at one or more loci, N_al3a_: number of individuals with at most three alleles at one or more loci, N_al4a_: number of individuals with at most four alleles at one or more loci. ^#^ Ploidy levels were inferred by allele sizing from fluorescent electropherograms in STRand [34] and confirmed by means of simulation (see Fig. S1 and Fig. S2). Hs: estimates of gene diversity averaged over loci and rHs: estimates of gene diversity averaged over loci and computed on a rarefied number of 12 haploid genomes in Adegenet [38] in R v. 2.12.0 [36].

### DNA Isolation and Microsatellite Typing

Total genomic DNA was isolated from 20 mg of silica dried *A. glutinosa* leaf tissue, ground on a Tissue Lyser II mixer mill (Qiagen) with one 3 mm-diameter tungsten bead (Qiagen) following two disruption cycles of 60 s each, using Isolate Plant DNA mini kit (Bioline) according to manufacturer instructions and stored at −20°C until further use. The 551 individuals were subsequently genotyped at twelve independent microsatellite loci, assumed to evolve neutrally, described in full in [32], namely, Ag01, Ag05, Ag09, Ag10, Ag13, Ag14, Ag20, Ag23, Ag25, Ag27, Ag30, Ag35, amplified in one single multiplexed-PCR according to the protocol described in [32]. In short, 12Plex PCRs were carried out in a final volume of 5 µL using 1X Type-it Microsatellite PCR Master Mix (Qiagen), with unequal concentrations of each of the 12 fluorescent forward primers labelled with one of 6-FAM, VIC, PET or NED dyes and reverse unlabelled primers (see [32]) and 5 ng of template DNA. PCR cycles were performed on a Veriti thermal cycler (Applied Biosystems) and consisted of an initial denaturation step of 5 min at 95°C, followed by 30 cycles of 95°C for 30 s, 58°C for 180 s and 72°C for 30 s and a final elongation step of 30 min at 60°C. PCR products were sent for fragment analysis on an ABI3730xl capillary sequencer to DNA Sequencing and Services (Dundee, UK) with a 1∶50 dilution before run and using GeneScan 500 LIZ internal size standard (Applied Biosystems).

The resulting fluorescence profiles were sized in STRand [34] jointly with the ones previously obtained from 36 *A. glutinosa* individuals (for details see [32]) sampled in a protected SSSI (Site of Special Scientific Interest) designated alder-dominated floodplain in Scotland which to the best of our knowledge has not been genetically disturbed by human-activities (Fig. 1). The inclusion of this ancient European population (referred to as PGM- for Post Glacial Maximum -hereafter) acts as a time-anchor to coalescence-based demographic scenario testing (see below) necessary to clarify the evolutionary history of the species in North Africa. The choice of this population which lies at the Northern range of the core distribution (Fig. 1) as a time-anchor is suitable because its location is Southern enough that the population does not belong to the current Northern leading-edge but is Northern enough that we know with certainty that the area has been recolonised from more Southern refugia not earlier than 8000 BP [15], [29] which serves as the time-anchor in demographic scenario testing below.

Sized peaks were exported to the MsatAllele package [35] in R v. 2.12.0 [36] to allocate peaks to suitable allele bin range. 93 individuals (16.8% of the total) were included in a repeat blind independent amplification and fragment analysis test in order to quantify genotyping error rates due to allelic dropout (E1), and to other genotyping error (E2) in Pedant V1.0 [37].

### Statistical Analyses

#### Genetic diversity

Unexpectedly, individuals from the four Moroccan populations exhibited up to four alleles at each of the 12 loci genotyped suggesting a difference in ploidy level between populations sampled in Morocco and those sampled elsewhere (Fig. S1 and results section below). In order to quantify levels of genetic variation within populations and compare them between populations with different ploidy levels, Nei’s gene diversity was estimated within each population and averaged over loci using the estimator Hs in Adegenet [38] in R. In addition, to account for differences in sample size and ploidy levels we subsample 12 haploid genomes at random from each population to compare the genetic diversity obtained after rarefaction (noted rHs). Differences in Hs and rHs between countries were tested using a Kruskal-Wallis rank sum test in Agricolae v.1. 1–2 (de Mendiburu, the International Potato Centre, 2012) in R.

#### Genetic differentiation

Regional genetic structure was first described using the individual-based Bayesian genetic assignment method implemented in Structure v.2.3.3 [39], [40], [41] to infer the number and distance between distinct genetic clusters (*K*) formed by the 587 individuals sampled in North Africa and in PGM with no *a priori* partitioning of individuals into groups defined according to their geographic location because such partitioning may not reflect true panmictic populations and may affect both estimates of genetic structure and their interpretation [42].

We followed the instructions provided in the Structure user guide [43] closely to use the appropriated model and parameters and to code the diploid and tetraploid genotypes in a way that allow the software to account for allele copy number in tetraploid genotypes and handle a mixture of diploid and tetraploid multilocus genotypes. We account for genotypic ambiguity in tetraploid individuals by using the *Recessive Alleles* model [41]. We coded partial tetraploid (ambiguous) genotypes by replicating one or two of the observed alleles to code the individual genotype at one locus with four named alleles. For instance, if three alleles (A, B and C) were observed, the genotypes was coded as A, B, C, C. We set the “recessivealleles” parameter to 1, the missing data to −5, the ploidy number to 4, the code “notambiguous” to −1, an integer that does not match any of the alleles in the data and that is different from the missing data code (−5) and the recessive allele code to −5 (missing data) at the top of the input file. This combination of settings indicates that all loci have ambiguity in allele copy number for tetraploid individuals. Finally, diploid individuals were coded with their two alleles followed by two missing coded (either A, A, −5, −5 for homozygotes or A, B, −5, −5 for heterozygotes) indicating to Structure that these genotypes are diploids.

The most-likely number of genetic clusters (K) was determined inspecting the variation of the mean logarithm of the probability of the data (Ln P(D)) across replicated runs. We ran 20 independent runs for K = 1 to K = 10 using a burn-in period of 50,000 steps followed by 100,000 Markov Chain Monte Carlo iterations, with an *Admixture* model of ancestry (parameter α inferred from the data using a uniform prior distribution initialized at 1) which allows for individual to have mixed ancestry and a *Correlated Allele Frequency* model (parameter λ set to 1 and not assuming same F_st_ among populations) which assumes that populations have each undergone independent drift away from a hypothetical ancestral population [40]. We then used the online tool Structure Harvester [44] to compute and plot the mean and variance of Ln P(D) again the range of K values. Following [45], we determined the best number of clusters K as the one that allows for Ln P(D) reaching a plateau where an additional increase of K value do not provide much increase in the likelihood. The matrix of genetic distance (allele frequency divergence) between the inferred clusters provided by Structure was used to build a neighbour joining tree using the ape package in R [46].

In order to validate those results, regional genetic structure was then described using a multivariate statistics based clustering method that requires fewer assumptions than population genetics model based methods such as Structure. We used a discriminant analysis of principal components (dapc, [47]) implemented in the adegenet package for R [38] that is well-suited to detect and analyse population genetic structure in complex genetic datasets such as those containing a mixture of different ploidy levels [48]. We first used the Polysat package [49] to compute Lynch genetic distance matrix [50] between individuals. The following steps were performed using adegenet. We first used the *find.clusters* function to transformed the original data using a principal component analysis (PCA) while keeping all principal components to maximize the variation. A k-means clustering was then applied to identify the best number of clusters K that minimize the variation within clusters using Bayesian Information Criterion (BIC). A discriminant analysis (DA) was then applied using the *dapc* function using 30 PCs explaining more than 90% of the total variance of the data and retaining all discriminant functions (equal to the best number of clusters, K, minus 1) and membership probabilities of each individuals to each clusters was then computed. The relationship between clusters was analysed building a neighbour joining tree based on the dapc distance between centroids of the different clusters with the ape package [46].

#### Historical demographic scenario choice and parameter estimation

**Figure 2:**
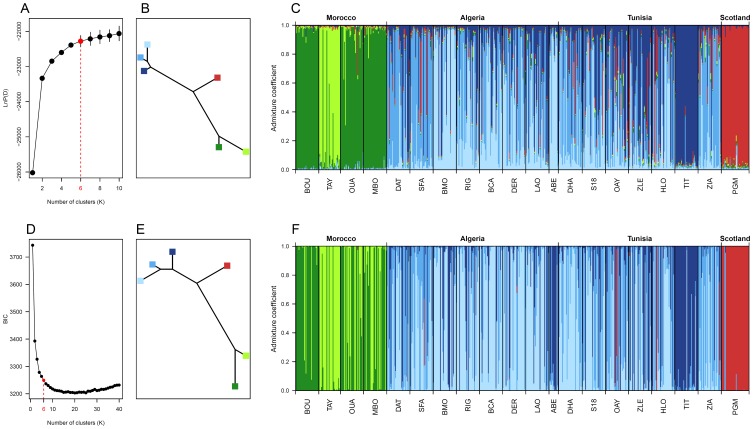
Regional genetic structure of *A. glutinosa* as inferred by Structure (A, B and C) and DAPC (D, E and F). Results from the population genetics model-based Bayesian clustering method implemented in Structure: (A) variation of the likelihood of the data across a range of number of clusters K; (B) neighbour joining tree computed using the genetic distance (allele frequency divergence) between clusters; (C) histogram of individual assignment to clusters where each individual is represented by a thin vertical bar partitioned into several coloured segments proportionally to its membership of a given cluster (admixture coefficient). Results from the multivariate statistics based clustering method implemented in DAPC: (D) variation of the Bayesian Information Criterion (BIC) of the k-means clustering algorithm across a range of number of clusters K; (E) neighbour joining tree computed using the DAPC distance between centroids of the clusters; (F) histogram of individual assignment to clusters where each individual is represented by a thin vertical bar partitioned into several coloured segments proportionally to its membership of a given cluster (admixture coefficient). All graphs were plotted in R v.2.12.0 [36].

The Approximate Bayesian Computation (ABC) method implemented in diyabc v.1.0.4.46beta [51] was used to explore likely scenarios of demographic history that may have generated current regional genetic structure and to estimate associated demographic parameters. Due to computational limitations and infinity of possible scenarios when numerous populations are considered, inferences were based on a subset of three standing populations, namely BOU (Morocco), TIT (Tunisia) and PGM (Scotland), representative of the three regional clusters determined by individual genetic assignment in structure and dapc but showing limited admixture (Fig. 2) thus respecting assumption of population isolation made by diyabc.

**Figure 3:**
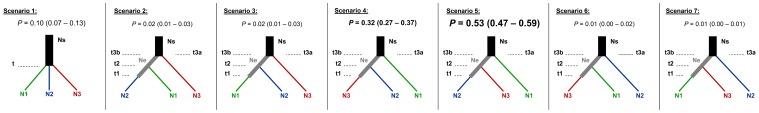
Representation of seven tested scenarios of past population history of north-African *A. glutinosa*. Scenarios were tested by means of Approximate Bayesian Computation (ABC) in diyabc [51] and based on genotyping of 96 individuals at 11 microsatellite markers sampled in three populations from Morocco (BOU, of effective size N1), Tunisia (TIT, of effective size N2) and Scotland (PGM, of effective size N3) representative of the regional genetic structure and identified as genetically distinct with no admixture in structure (Fig. 2). Ne and Ns refer to effective size of ancestral populations and t, t3a,b, t2, t1 to divergence times. Posterior probabilities (*P*) of the scenarios and 95% confidence intervals of *P* (in brackets) were obtained by means of simulations. 7.10^3^ out of 70.10^6^ simulations closest to the real genetic dataset were subjected to a weighted polychotomous logistic regression to estimate P [33].

Seven demographic scenarios characterised by divergence times in generations (t, t1, t2, t3a and t3b), effective population size of standing (N1, N2 and N3 for BOU, TIT and PGM respectively) and putative ancestral (Ne and Ns) populations were compared (Fig. 3). In the simplest scenario (scenario 1, Fig. 3), all three populations diverged simultaneously at time t from a common ancestor. Scenario 2 to 7 consider all possible dichotomous scenarios of population divergence (Fig. 3) where a population diverged from the ancestor (of effective size Ns) at time t3a while the other two populations diverged more recently from a common ancestor (of effective size Ne and that originated from the previous ancestor at time t3b) respectively at time t2 and t1 (Fig. 3).

For each simulated scenario, priors were set as a wide Uniform distribution bounded between 10 and 20,000 individuals for N1, N2, N3, Ne and Ns and between 1 and 20,000 generations for divergence times with the additional constraint of t1<t2<t3a,b. Default values were used for genetic parameters, assuming a Generalized Stepwise Mutation model [52] for microsatellite mutation, using a Uniform prior distribution bounded between 10^−3^ and 10^−4^ mutation per haploid genome and generation (*µ_SSR_*∼U[10^−4^–10^−3^]) while using a Uniform prior distribution bounded between 0.1 and 0.3 for P (*P*∼U[0.1–0.3]), the parameter of the Geometric distribution in which the number of repeats of the mutation is drawn. Within this general parameterisation of the mutation model, mutation parameters were allowed to vary independently for individual loci using a hierarchical scheme to account for heterogeneity in mutation rate and model across loci (using Gamma distributions: *µ_SSR_*∼G[10^−5^–10^−2^, 2]; *P*∼G[0.01–0.9, 2] and allowing for point mutation in the flanking sequence *µ_SNI_*∼G[10^−9^–10^−3^, 2]).

Input data consisted of diploid multilocus genotypes scored at 11 microsatellite markers for 30 (TIT) to 36 (PGM) individuals per population which are used by diyabc to compute observed summary statistics. In order to include information content from the 30 tetraploid individuals sampled in BOU in analyses, allele frequencies at each locus for BOU were first estimated using the function simpleFreq in the R package polysat [49] and used with the function sample in R to generate 30 diploid multilocus genotypes assuming random mating as input data for diyabc to generate equivalent summary statistics.

The observed and simulated genetic datasets were summarised using mean number of alleles, mean size variance and M index [53] for each population and for each pair of populations, mean number of alleles, mean size variance, classification index [54], Fst [55] and shared allele distance [56] between populations.

70.10^6^ simulations were run providing 10.10^6^ simulations for each scenario. The 7,000 simulations closest to the real genetic dataset were subjected to a weighted polychotomous logistic regression [33] to estimate posterior probabilities (P) for each scenario. Confidence in scenario choice was assessed by simulating 1,000 datasets for each scenario and assigning the most likely scenario to each of these dataset. Following [57], type I error of wrongly accepting a false scenario was computed for a particular scenario as the proportion of simulated scenarios generated under the focal scenario that support other scenarios, and type II error of wrongly rejecting a true scenario as the proportion of datasets simulated under all other scenarios that was assigned to the focal scenario.

Demographic parameters were inferred from the most-likely scenario using the normalized Euclidian distance between the observed and simulated summary statistics to select the 10,000 simulated datasets closest to observed data (0.1% of the simulations). Parameters’ posterior distributions were estimated using a local linear regression method [58] and plotted in R using the package locfit [59]. The performance of parameter estimation was assessed by simulating 1,000 pseudo-observed datasets generated using known demographic parameter values drawn from prior distributions. Comparisons of known and estimated demographic values were used to infer bias and precision of estimations [33].

## Results

### Microsatellite Typing

A total of 587 *Alnus glutinosa* individuals were genotyped at 12 microsatellite loci across 20 populations (Table 1). As previously observed [32], AG14 showed a high rate of missing genotype (36.5%, 214 missing genotypes out of 587) and allelic dropout error (E1 = 58.1% [37.9%–75.5%]) indicative of null allele occurrence and was removed from subsequent analyses. The remaining 11 loci showed a low rate of missing data (0.19%, 12/6457 genotypes; available via Dryad, doi:10.5061/dryad.3801d). Repeated blind genotyping of 93 individuals (16.8% of the total) gave very low genotyping error with a mean allelic dropout (E1) across loci of 0.24% (sd: 0.28%, min: 0.00% [0.00%–0.97%], max: 0.66% [0.01%–4.35%]) and no other kind of genotyping error (E2) detected (E2 95% CI from [0.00%–0.83%] to [0.00%–1.16%] across loci).

### Ploidy Level and Genetic Diversity

Unexpectedly, up to four alleles per locus were genotyped in individuals from the four Moroccan populations (Fig. S1) with four alleles detected at, at least, one locus (Table 1) for most individuals and an average of 30 alleles across 11 loci (min: 22 and max: 38 alleles; Fig. S2). However, for one individual with 50% of missing data, no more than two alleles per locus were detected across loci and only 12 alleles over the six genotyped loci (Table 1 and Fig. S2). The high number of alleles observed in the Moroccan populations contrasted sharply with all other populations in which only one or two alleles were detected at every locus (Table 1) with an average of 17 alleles across 11 loci (min: 13 and max: 21 alleles, Fig. S2). The high number of alleles within Moroccan individuals clearly indicates that these populations consist of tetraploid individuals as confirmed by simulations (Fig. S2). It should be noted that this polyploidisation event concerns exclusively individuals from Morocco, analysed and genotyped blindly according to geographic location altogether with individuals sampled in Algeria and Tunisia.

Overall mean gene diversity in North African populations of alder was relatively low for a tree species with Hs ranging for 0.48 to 0.78 (mean: 0.59, sd: 0.10; Table 1). Genetic diversity estimated for equal genome sampled size (rHs) ranged from 0.47 to 0.76 (mean: 0.57, sd: 0.09; Table 1) and was significantly different between countries (Kruskal-Wallis H = 12.7, df = 2, *p*-value = 0.0017) with Morocco showing the highest mean gene diversity (rHs = 0.72) followed by Tunisia (rHs = 0.55) and Algeria (rHs = 0.50).

### Regional Genetic Structure

The log-likelihood of the probability of the data as computed by the population genetic clustering based model implemented in Structure reaches a plateau for a number of cluster K = 6 (Fig. 2A). While the likelihood gain is substantial between K = 5 and K = 6, it is minimal for higher value of K. Note that using the hierarchical Evanno’s delta K method [60] provides similar most-likely number of cluster and that choosing a number of cluster of K = 5 or K = 7 does not result in different pattern of detected population genetic structure (results not shown). Three closely related clusters (Fig. 2B) characterise Algerian and Tunisian populations (Fig. 2C), two closely related genetic clusters are found in Moroccan populations (Fig. 2C) while PGM population is composed of one genetic cluster (Fig. 2C). Unexpectedly, the PGM cluster shows an intermediate genetic distance with those representing both sides of the studied area in North Africa (Fig. 2B), with the group formed by Moroccan populations on one side and the one formed by Algerian and Tunisian populations on the other side.

Results from the multivariate statistical approach mostly confirm the regional genetic structure pattern observed in Structure (Fig. 2D, 2E and 2F). Namely, the BIC variation across a range of number of cluster K identify K = 6 as a likely partition of the structure in the genetic data. Note that as previously, choosing a smaller or higher number of cluster does not change the general pattern of genetic differentiation detected but instead groups together closely related clusters or subdivides other clusters respectively (results not shown) but never compromising the general pattern. Relative genetic relationship between clusters and cluster distribution across populations are very similar to results from Structure with PGM population being genetically intermediate between clusters characterising Moroccan and Tunisian/Algerian populations (Fig. 2E). Only slight differences in the cluster repartition among individuals could be observed between the two methods with DAPC showing more cluster mixture among Moroccan population (Fig. 2C) and Structure inferring more admixed individuals among Algerian/Tunisian populations. Consistency in the results obtained with the two different approaches is remarkable and demonstrate a robust and strong genetic structure that is independent of the analytical method used.

### Inference of Demographic History of North African *A. Glutinosa* Populations Using ABC

Among the seven tested scenarios, two demographic models, scenario 4 and 5 gave highest posterior probability from the data (Fig. 3). Common to both models, Tunisia and PGM share a recent common ancestor while Morocco diverged earlier. The difference between these models relates to the timing of divergence of the Tunisian populations and PGM from their common ancestor (Fig. 3), with highest support for recent divergence of Tunisia, scenario 5, with a higher posterior density (P) and no overlapping of 95%CI for P between the two most-likely scenarios (scenario 5: *P* = 0.53 (CI95% = 0.47–0.59); scenario 4: *P* = 0.32 (CI95% = 0.27–0.37)). Yet, in simulations made to access the power of our dataset to discriminate between alternative scenarios, one third of pseudo-observed datasets assigned to scenario 5 were simulated under scenario 4 (Table S1). This highlights the relative lack of power of our dataset to discriminate between closely related scenarios differing by recent divergence timing. However, wrong assignment to scenario 5 of pseudo-observed datasets simulated under scenario 4 is by far the most common error since pseudo-observed datasets simulated under scenarios other than scenario 5 are almost never assigned to scenario 5, thus giving strong support to earlier divergence of Morocco (Table S1).

**Figure 4:**
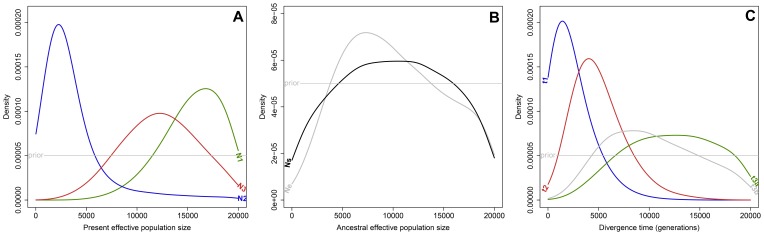
Demographic parameter posterior densities: contemporary (A) and ancestral effective population sizes (B), divergence times (C). Estimates resulted from the most-likely scenario 5 (Fig. 3) of population history of *A. glutinosa* estimated in diyabc [51] based on genotyping of 96 individuals from three genetically distinct isolated populations of Morocco (BOU), Tunisia (TIT) and Scotland (PGM). See Fig. 1 and Fig. 3 for details. Divergence times are expressed in generations and effective population sizes in number of diploid genomes.

Demographic parameters were estimated under the most-likely scenario, scenario 5. Estimates of effective population size were contrasted across population (Fig. 4A) with TIT much smaller (N2∶1620 [898–10500] diploid genomes, median and 5–95% quantiles of the posterior distribution respectively, Table 2) compared to PGM (N3∶12100 [6230–18400] diploid genomes and BOU (N1∶16600 [10100–19500] diploid genomes). Note that BOU effective population size expressed in number of tetraploid genomes (8800 [5050–9750]) fall in between TIT and PGM effective population size. Estimates of effective population size for ancestors were moderate (Ne: 7140 [2770–18700], Ns: 8680 [1550–18600]; Table 2) with relatively flat posterior distribution (Fig. 4B) and relatively high bias and error and low Factor 2 (Table S2) indicative of relatively low information content of the data to estimate these parameters precisely. Consistent with scenario 5, divergence of TIT from its ancestor was estimated to be slightly more recent than that of PGM (Fig. 4C and Table 2; t1∶709 [181–6120] and t2∶3880 [1850–9830] generations, respectively). Although mean relative bias for these estimates is low, high error and low Factor 2 for t1 (Table S2) together with wide and overlapping credible interval for the divergence times and relatively high support for scenario 4 render determination of the relative timing of divergence between PGM and TIT from their common ancestor difficult. Divergence time estimates for BOU (t3a: 14400 [4930–19000] generations, Table 2) and for the common ancestor of TIT and PGM (t3b: 8930 [3700–18300] generations, Table 2) seems to be better estimated although with a slightly positive bias (Table S2), the latter being slightly more recent than the Moroccan divergence. Assuming a generation time of 10 years for *A. glutinosa*, divergence times translate to 7090 [1810–61200] years for TIT, 38800 [18500–98300] PGM and 144000 [49300–190000] years for BOU.

**Table 2:** Characteristics of prior and posterior distributions of demographic parameters of population history of *A. glutinosa* estimated under the most-likely of seven tested scenarios by means of Approximate Bayesian Computation in diyabc [51].

	Prior distribution			Posterior distribution		
Demographic parameter#	Family	min	max	mode	Q_0.05_	Q_0.95_
N1	Uniform	10	20000	16600	10100	19500
N2	Uniform	10	20000	1620	898	10500
N3	Uniform	10	20000	12100	6230	18400
Ne	Uniform	10	20000	7140	2770	18700
Ns	Uniform	10	20000	8680	1550	18600
t1	Uniform	1	20000	709	181	6120
t2	Uniform	1	20000	3880	1850	9830
t3b	Uniform	1	20000	8930	3700	18300
t3a	Uniform	1	20000	14400	4930	19000

# Details of demographic scenarios tested and estimated parameters are given in Fig. 3 and Fig. 4. N1, N2, N3a and N3b, Ne, Ns refer to effective population sizes in number of diploid genomes respectively of standing populations from Morocco (BOU), Tunisia (TIT) and Scotland (PGM) and from ancestral populations from which they have diverged as modelled in the most-likely of the seven tested scenarios (Fig. 3). t1, t2, t3a and t3b: divergence time of the standing populations from the ancestral populations in generations (Fig. 3). Q_0.05_ and Q_0.95_∶5% and 95% quantiles.

## Discussion

The present empirical study reveals strikingly contrasted demographic history of *A. glutinosa* at the rear-edge of the species’ distribution in North Africa. The originality of the study lies first in its large-scale sampling across the Mediterranean fringe of North Africa of previously poorly described populations. These populations inhabit barely accessible landscapes in small pockets of locally wet habitat in a regionally thermo-Mediterranean climate characterised by dry summers. This study is also original in its analysis of recently developed nuclear microsatellite markers using state-of-the-art methodology for quantitative demographic history inference. In doing so, new insights were gained into the evolutionary features of rear-edge populations of a riparian keystone tree species.

### Contrasted Rear Edge Populations

North African *A. glutinosa* populations are located at the Southern tip of the contemporary core distribution area of the species, in a scattered, patchy distribution, isolated from the main distribution area that stands across continental Europe (Fig. 1). With such a peculiar biogeographical setting, *a priori* expectations would be that these populations are climate relicts that have persisted since the last glacial period or even longer.

This expectation is supported by fossil pollen records showing a continuous presence of the species, all be it with various pollen abundance depending on the period, in Tunisia since 60,000–50,000 cal. BP [26], [27], in Algeria since 20,000 cal. BP [28], and in Morocco since 10,000 cal. BP [30]. However, the regional genetic structure of the 19 populations sampled in North Africa and its comparison with the one of a known postglacial population sampled in Scotland within the European range of the species’ distribution tells a more complex story. First, Tunisian and Algerian *A. glutinosa* populations are more closely related to the PGM population than to Moroccan populations indicating that North-African populations at the two ends of the South Mediterranean basin have diverged for a long-time without any connection or gene flow. This is clearly shown by the ploidy difference between the two North-African regions and the relatively long distance between genetic clusters and the clear-cut genetic clustering location within each region. Remarkably, and unexpectedly, the only genetic cluster that spread across regions is the one defined by PGM samples found at low percentage in Algerian and Tunisian populations (Fig. 2C and F). Such a pattern may indicate that these Southern populations may have acted as LGM refugia that participated to the recolonisation of Europe. This hypothesis may also find some support from the geographical trend in genetic diversity that is lower in Southern populations (Algeria and Tunisia) compared to Northern populations (PGM), the latter probably resulting from admixture with populations originating from other refugia located in central or eastern Europe [12]. Hypothesis testing by approximate Bayesian computation does not best support this interpretation (scenario 4) but supports PGM to be older (t2∶3880 [1850–9830] generations) than TIT (t1∶709 [181–6120] generations]). This information suggests the interpretation of a transient population dynamic, with more recent and less diverse local populations, in the region defined by Algeria and Tunisia. Whilst a finer scale analysis of populations in Algerian, Tunisian and southern Europe would help clarify the situation for north-eastern populations, we have a clear indication of the distinctiveness of Moroccan populations.

### High Distinctiveness of Relict Populations

Indeed, Moroccan populations were estimated to be ancient relicts with a divergence time estimated at 14400 [4930–19000] generations. These populations are characterised by a high genetic diversity and a strong genetic distinctiveness compared to the other populations studied with, for the first time, the identification of natural tetraploids for this species. Although artificial tetraploids have been obtained by colchicine treatment [61], the species was to our knowledge until now only reported as a 2n = 28 diploid species across its entire distribution area.

Tetraploid individuals usually originate from fertilisation involving unreduced gametes that can be due to several causes [62]. The first mechanism involves autopolyploidy though chromosome doubling within an individual. The second is autopolyploidy though hybridization between differentiated populations within a species. The third scenario is allopolyploidy by interspecific hybridization. We clearly have no data to differentiate autotetraploidy from allotetraploidy here as this necessitates studying allele segregation within progenies to distinguish between disomic and polysomic inheritance typical of allopolyploids and autopolyploids, respectively [63], [64]. On the other hand, allopolyploidy by interspecific hybridization appears unlikely because the Moroccan population are unequivocally not assigned by Structure (even partially) to closely related *A. incana* or *A. cordata* species (results not shown) and the genetic distance between the tetraploid Moroccan populations and the diploid populations is within the same magnitude that the genetic distance found between Scottish and Tunisian/Algerian diploid populations. Given the high genetic diversity within tetraploid populations, a scenario of autopolyploidy by hybridization between differentiated *A. glutinosa* populations seems more likely than a chromosome doubling within individual. Indeed the high genetic diversity standing in tetraploid population is not due only to the numeric increased in genome number alone as genetic indices computed on a rarefied sampled size still show a statistically significantly higher genetic diversity within Moroccan populations (Table 1). An ancient hybridization event merging the genomes of differentiated populations resulting in an additive effect on genetic diversity may have increase within-individual genetic diversity that was then maintained though time in tetraploids. In addition, such an event may account for the much higher effective population size estimate in the Moroccan population by the coalescent approach in spite of the low number of individuals still standing on these small and isolated populations (Table 2). The geographical location of the Moroccan populations, close to the Gibraltar Strait, at the crossroad between the African and Eurasian continents, is a likely area for secondary contact and hybridization between diverged populations [65]. One hypothesis for the emergence of tetraploids may be the hybridization between ancestral North African and ancestral European populations close to the Gibraltar Strait at some point in time [66], [67].

Regardless of the cause for tetraploidy in Morroccan *A. glutinosa*, it is noticeable that only tetraploids were found there suggesting that perhaps only tetraploids survived because they were better adapted to fluctuating environmental pressure in extremely arid habitats [68]. Factors that may explain the successful establishment of tetraploids are numerous especially in the context of strong selection pressure caused by environmental fluctuation [68], [69]. In the short term, polyploidization especially when caused by hybridization between divergent populations or species, can cause quick change in gene expression and increase phenotypic plasticity, which may be beneficial for survival to an environmental crisis or fast environmental change [70]. Furthermore, the higher genetic diversity found in polyploid populations can decrease the effect of inbreeding depression in small populations [71], an immediate effect that can give substantial advantage for the establishment of polyploid individuals. In the long term, genetic redundancy caused by chromosome doubling allows exploring new evolutionary trajectories because the functional pressure imposed by selection is relaxed in the duplicated gene copy which is freer to mutate giving rise to new or complementary functions [69], [72]. In addition, as genetic diversity is increased by genome doubling, genetic drift is reduced and more functional alleles are potentially exposed to natural selection efficiency increases allowing for the population to adapt more efficiently [72]. Such genome flexibility could explain the long-term persistence of *A. glutinosa* as a climate relict in Morocco.

The relict nature and the striking distinctiveness of Moroccan *A. glutinosa* populations legitimate a high priority conservation status for the region given on-going climate change. These populations have been able to survive various climatic and environmental changes throughout at least the last glacial cycles. Polyploidization has probably played an important role in this adaptability and in maintaining high genetic diversity by increasing the effective population size [71]. The original polyploidy of these populations seems to go with particular phenotypic features with for instance, the distinct leave morphology we observed in these populations (data not shown). As *A. glutinosa* is an important keystone species shaping the whole riparian ecosystem (i.e. foundation species), the high distinctiveness of these relict populations may be accompanied with distinctiveness of the whole dependent ecosystem. Indeed, series of studies in community genetics initiated in the riparian ecosystem shaped by cottonwoods (*Populus* spp.) have demonstrated that foundation species genetics have impact not only for the species phenotype but also for the community and/or ecosystem phenotype [73]. It is thus very likely that interspecific interaction in these populations have been built on very distinct genetic basis as in typical riparian ecosystem shaped by diploid *A. glutinosa*. The high ecological and evolutionary values of these *A. glutinosa* populations may thus extend beyond the tree species to the whole community standing there probably as relict ecosystem that persisted throughout the ages.

### Conclusion

Overall our study illustrates the complexity of rear-edge population history [5], [6] that includes more recent populations more closely related to the contemporary core distribution area, and ancient relict populations that persisted though times thanks to a combination of ecological (riparian ecosystem [10]), biological (polyploidization [69]) and geographical (the rough topography including deep valley and Atlantic influence of the Gibraltar Strait region [66]) characteristics that contributed to their possible resilience to past climate changes. Long-term persistence and stability of relict populations in the Mediterranean region has been demonstrated for a number of taxa [74] pointing out the invaluable conservation value of the region [31] and urging policy to prioritise conservation in the Mediterranean region given ongoing political and expected considerable climatic and anthropogenic pressures on its standing biodiversity [74].

## Supporting Information

Figure S1 Examples of microsatellite electropherograms leading to categorisation of individuals as diploids or tetraploids.
(PDF)

Figure S2 Comparison of observed and expected distribution of the number of alleles per individual by ploidy level.
(PDF)

Table S1 Power to discriminate between alternative demographic scenarios tested by coalescence.
(PDF)

Table S2 Confidence and precision of parameter estimates under the most-likely of seven tested scenarios of population demographic history.
(PDF)
