# Supplementary material for: High Genetic Diversity and Distinctiveness of Rear-Edge Climate Relicts Maintained by Ancient Tetraploidisation for Alnus glutinosa
Source: PLoS One. 2013 Sep 30;8(9):e75029. doi: 10.1371/journal.pone.0075029 (PMC3787099; doi:10.1371/journal.pone.0075029)

Supporting Figure S1: Example of fluorescent electrophoretic profiles for diploid (a, b) and tetraploid (c, d) *A. glutinosa* individuals genotyped at 12 microsatellite markers described in Lepais & Bacles (2011). Sized alleles are annotated with squares; size standard peak sizes are indicated above each corresponding peak. Locus names are shown in (a). Note Ag14 is missing in these four individuals probably due to presence of null alleles

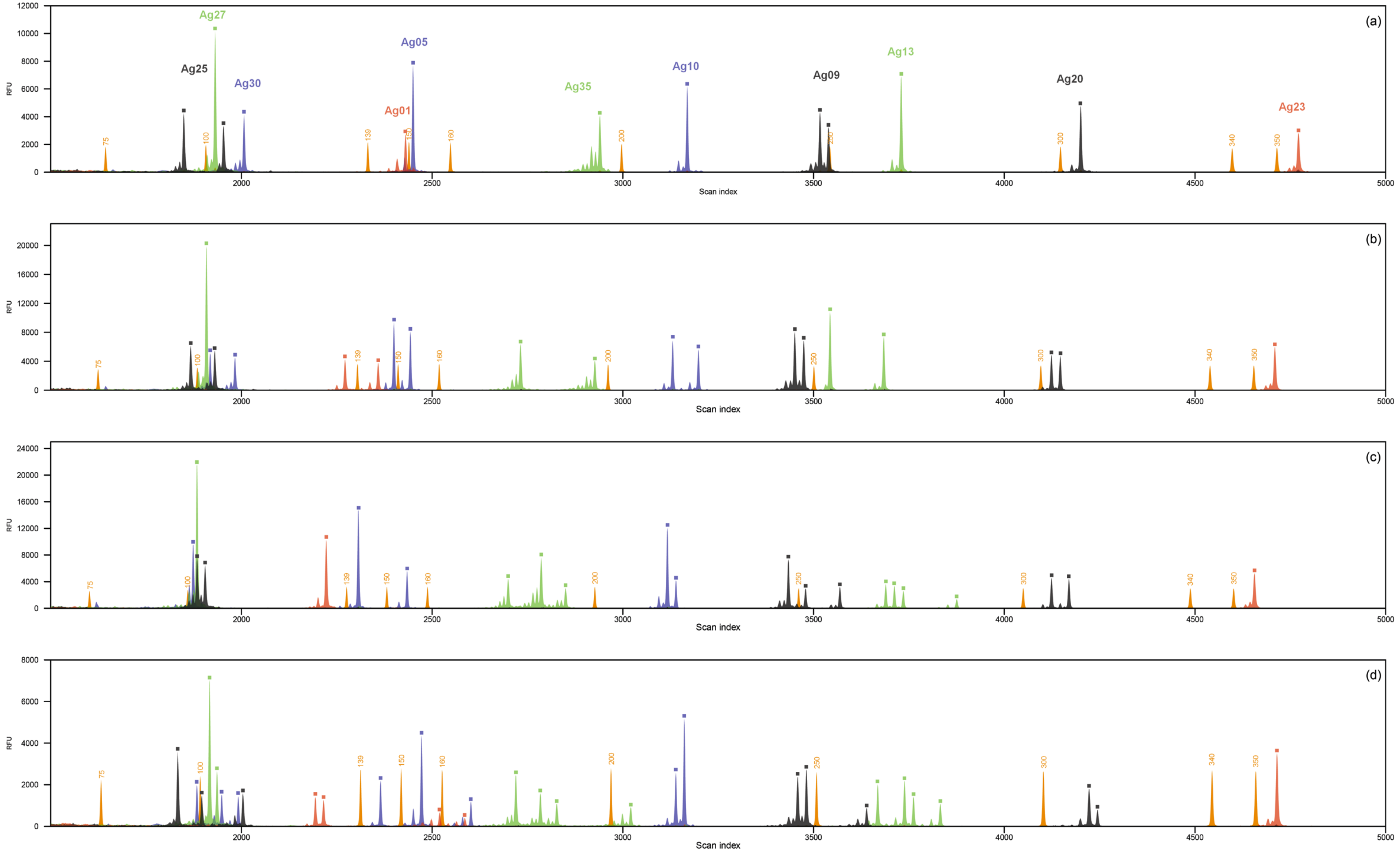

Supplement: Figure S1 — Examples of microsatellite electropherograms leading to categorisation of individuals as diploids or tetraploids. (PDF) [file pone.0075029.s001.pdf]
