# Supplementary material for: High Genetic Diversity and Distinctiveness of Rear-Edge Climate Relicts Maintained by Ancient Tetraploidisation for Alnus glutinosa
Source: PLoS One. 2013 Sep 30;8(9):e75029. doi: 10.1371/journal.pone.0075029 (PMC3787099; doi:10.1371/journal.pone.0075029)

Supporting Figure S2: Number of alleles within individuals observed in Moroccan (red, N=118) and all other (blue, N=469) natural populations based on genotyping of at 11 independent microsatellite loci (described in Lepais & Bacles 2011). Distribution of the number of alleles expected in an equivalent sample of randomly mating diploid (dark grey) triploid (light grey) and tetraploid (black) individuals based on simulated datasets of 500 individuals drawn from alleles frequencies estimated from 487 genotyped samples and assuming linkage equilibrium Distributions were found to differ significantly (Kruskal-Wallis H=1768.7, df=4, *p*-value<0.001; pairwise comparisons: *p*-values<0.001) with the largest mean difference of 13 alleles found between the distribution of observed alleles in Morocco and of observed alleles in all other sampled populations. Note that Moroccan populations show a slightly higher number of alleles compared to the simulated tetraploids and conversely, simulated diploids have a higher number of alleles that observed diploids in natural population. This effect is due to the fact that simulated genotypes were created using allele frequencies averaged over all populations which as a result averages the genetic diversity of the simulated genotypes (that was hence lower for simulated tetraploids compared to the highly diverse Moroccan populations, and conversely for diploid individuals).

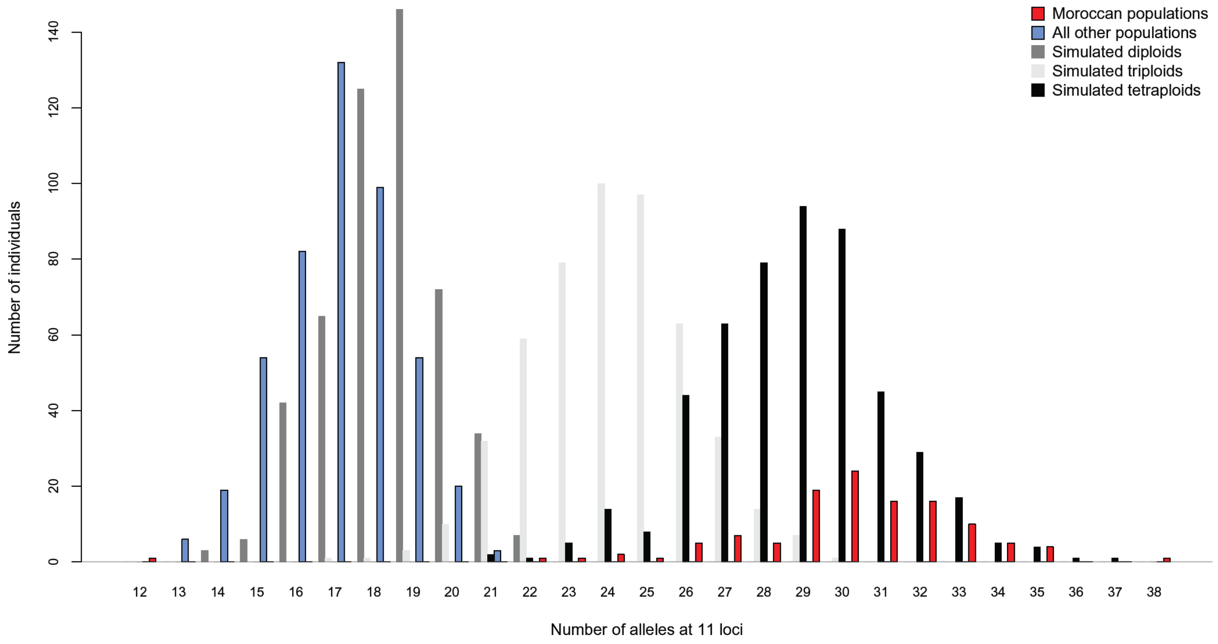

Supplement: Figure S2 — Comparison of observed and expected distribution of the number of alleles per individual by ploidy level. (PDF) [file pone.0075029.s002.pdf]
