# Supplementary material for: High Genetic Diversity and Distinctiveness of Rear-Edge Climate Relicts Maintained by Ancient Tetraploidisation for Alnus glutinosa
Source: PLoS One. 2013 Sep 30;8(9):e75029. doi: 10.1371/journal.pone.0075029 (PMC3787099; doi:10.1371/journal.pone.0075029)
Supplement: Table S1 — Power to discriminate between alternative demographic scenarios tested by coalescence. (PDF) [file pone.0075029.s003.pdf]

**Supporting Table S1:** Power to discriminate between alternative scenarios of population history of *Alnus glutinosa* tested by coalescence.

| Simulated \ Most-likely | 1 | 2 | 3 | 4 | 5 | 6 | 7 | N (selected) | Type I error |
|---|---|---|---|---|---|---|---|---|---|
| 1 | **527** | 5 | 1 | 1 | 8 | 39 | 9 | 410 | 0.11 |
| 2 | 9 | **715** | 3 | 0 | 1 | 0 | 0 | 272 | 0.02 |
| 3 | 10 | <u>334</u> | **69** | 1 | 8 | 0 | 1 | 577 | 0.84 |
| 4 | 32 | 3 | 0 | **43** | <u>379</u> | 3 | 0 | 540 | 0.91 |
| 5 | 19 | 5 | 0 | 5 | **667** | 0 | 0 | 304 | 0.04 |
| 6 | 27 | 0 | 0 | 0 | 1 | **497** | 16 | 459 | 0.08 |
| 7 | 24 | 0 | 1 | 0 | 1 | 64 | **290** | 620 | 0.24 |
| Type II error | 0.19 | 0.33 | 0.07 | 0.14 | 0.37 | 0.18 | 0.08 | | |

All simulations were run in DIYABC (Cornuet et al. 2010). Confidence in scenario choice was assessed by simulating 1,000 datasets for each scenario and assigning the most likely scenario to each of these dataset. Following Bertorelle et al. (2010), type I error of wrongly accepting a false scenario was computed for a particular scenario as the proportion of simulated scenarios generated under the focal scenario that supports other scenarios, and type II error of wrongly rejecting a true scenario as the proportion of datasets simulated under all other scenarios that was assigned to the focal scenario. A scenario is assigned when the confidence interval of the probability of most-likely scenario does not overlap the confidence interval of the probability of other alternative scenarios, otherwise no scenario can be selected.
