# Supplementary material for: High Genetic Diversity and Distinctiveness of Rear-Edge Climate Relicts Maintained by Ancient Tetraploidisation for Alnus glutinosa
Source: PLoS One. 2013 Sep 30;8(9):e75029. doi: 10.1371/journal.pone.0075029 (PMC3787099; doi:10.1371/journal.pone.0075029)
Supplement: Table S2 — Confidence and precision of parameter estimates under the most-likely of seven tested scenarios of population demographic history. (PDF) [file pone.0075029.s004.pdf]

Supporting Table S2: Confidence and precision of parameter estimates under the most-likely of seven tested scenarios of population demographic history of *Alnus glutinosa*.

Demographic parameter	Prior distribution			Posterior distribution					Confidence in parameter estimation (mode)				
	family	min	max	mean	median	mode	$Q_{0.05}$	$Q_{0.95}$	Bias	RMSE	50% coverage	95% coverage	Factor 2
N1	U	10	20000	15500	15900	16600	10100	19500	-0.02	0.30	0.53	0.95	0.97
N2	U	10	20000	3530	2590	1620	898	8980	-0.03	0.52	0.51	0.96	0.88
N3	U	10	20000	12300	12300	12100	6230	18400	0.01	0.35	0.49	0.95	0.95
Ns	U	10	20000	10100	10100	8680	1550	18600	**0.78**	**11.77**	0.50	0.94	**0.58**
Ne	U	10	20000	10100	9640	7140	2770	18700	**0.32**	**2.59**	0.50	0.95	**0.67**
t1	U	1	20000	2270	1730	709	181	6120	-0.05	**3.56**	0.49	0.95	**0.21**
t2	U	1	20000	5030	4550	3880	1850	9830	-0.09	0.50	0.52	0.95	0.85
t3b	U	1	20000	10400	9960	8930	3700	18300	**0.31**	0.76	0.50	0.94	0.91
t3a	U	1	20000	12200	12200	14400	4930	19000	**0.33**	0.74	0.50	0.96	0.91

All simulations were run in DIYABC (Cornuet et al. 2010). Description of demographic parameters and tested population history scenarios are detailed in Figures 3 and 4. Bias: mean relative bias (distance between mode of the mode (global) and true value); RMSE: relative mean square error (precision, distance between each estimates ant the true value); 50% and 95% coverage: proportion of simulations in which the true value lies within the 50% and 95% credible interval around the estimate; Factor 2: proportion of estimated values falling within the interval between 50% and 200% of the true value. The performance of parameter estimation was assessed by simulating 1,000 pseudo-observed datasets generated using known demographic parameter values drawn from prior distributions. Comparisons of known and estimated demographic values were used to infer bias and precision of estimations (Cornuet et al. 2008).
